# The causal efficacy of consciousness: a neuroscientific analysis and explanation

**DOI:** 10.3389/fnhum.2026.1849369

**Published:** 2026-06-18

**Authors:** Chirapat Ukachoke

**Affiliations:** Neurology Center, Phyathai 3 Hospital, Bangkok, Thailand

**Keywords:** causal efficacy, consciousness, epiphenomenalism, hard problem, information, mental causation, neural circuit, qualia

## Abstract

This study analyzes neural and behavioral events occurring before, during, and after the emergence of a pain quale and the consciousness of that pain to examine the causal efficacy of the quale and consciousness. It finds that events occurring before or concurrently with the emergence of the quale are neither caused nor influenced by the quale or consciousness. Events occurring afterward may also be unaffected by the quale or consciousness if they do not overtly involve phenomenal content. By contrast, activities that explicitly incorporate phenomenal characteristics—such as experiencing, remembering, or reporting the pain quale—are influenced by the occurrence and phenomenal character of the quale or consciousness. Accordingly, qualia and consciousness are not inert. However, their influence does not operate through mechanistic particle–force interactions. Instead, they function as higher-level factors that stand in stable, constitutive counterfactual relations to downstream neural and behavioral effects. Physically, they perform this role by providing the involved neural causal chain with their phenomenal information, which is necessary in initiating and structuring that causal chain and determining the content of the outputs. Nevertheless, qualia and consciousness cannot generate these outputs by themselves; the relevant neural circuits must be present to implement the mechanistic processing required for producing physical effects. Only when both components function together is the causal chain complete and can yield results. Thus, the study concludes that qualia and consciousness influence the neural system and, although they are not mechanistic, independent, or complete causes of physical events, they can be considered causally efficacious in a restricted, information-based sense.

## 1 Introduction

The question of whether consciousness exerts causal effects—and, if so, how—is fundamental in both neuroscience and philosophy. Its resolution carries significant implications. If consciousness is causally inefficacious, major issues arise. Importantly, (1) neuroscience must explain how we can have experiences and knowledge of consciousness if it does not affect neural circuits; (2) evolutionary theory must account for the selection and persistence of a phenomenon without functional physical effects; and (3) at a personal level, we must answer why consciousness occurs in us and whether our lives could proceed as usual without it. Resolving this issue is therefore crucial.

In everyday life, this problem may not appear to arise. Consciousness appears to have causal power; it seemingly guides our thoughts and behaviors (Robb et al., 2023). However, this commonsense view is not indisputable. Empirical findings show that consciousness does not occur independently but arises from specific neural activity (Crick and Koch, 1998; Block, 2005; Dehaene and Changeux, 2011; Kandel and Shadlen, 2021). Since, according to our current neuroscientific knowledge, neural activity is fully embedded within the brain’s physical causal structure, it is possible that all effects attributed to consciousness are in fact produced by neural processes alone (see Kim, 2005, 2007). Moreover, because consciousness lacks particles and physical forces, it likely lacks the capacity to interact with a causally closed physical system (Chalmers, 1996, pp. 150–160; Robb et al., 2023; Walter, n.d.). These considerations raise the possibility that the apparent causal role of consciousness is illusory (Huxley, 1874; Jackson, 1982; Wegner, 2002; Pockett, 2004).

Historically, this issue has been framed in terms of epiphenomenalism, which holds that consciousness is produced by physical processes but exerts no causal influence (McLaughlin, 1989; Robinson, 2023; Walter, n.d.). Critics have raised numerous objections—appealing to, for example, counterintuitiveness, evolutionary considerations, knowledge of other minds, and self-stultification (Chalmers, 1996, pp. 196–197; Pauen, 2006; Megill, 2013; De Brigard, 2014; Baumeister et al., 2018)—while defenders have responded with various counterarguments (see Staudacher, 2006; Baysan, 2021; Rostek, 2023). However, in the absence of a widely accepted mechanism by which consciousness could exert effects, the matter cannot be settled, and the debate continues.

However, since the nineteenth century, empirical investigations related to this problem began to emerge. Although these studies were not designed to address the causal efficacy of consciousness directly, they have provided objective findings that bear significantly on the issue. Research on reflexes and conditioned responses (Hall, 1833; Boakes, 2023) and later studies on the timing of conscious intention and neural activity (Libet et al., 1983; Keller and Heckhausen, 1990; Haggard and Eimer, 1999; Soon et al., 2008) have advanced understanding of the relationship between consciousness and various neural reactions, behaviors, and motor initiation. Nevertheless, these findings do not directly address the question of consciousness’s causal efficacy.

Presently, although extensive philosophical investigation into this matter and empirical findings indirectly related to this issue already exist, neuroscientific analyses examining specifically whether and how consciousness is causally efficacious remain limited. The present study aims to address this gap by providing a theoretical analysis based on existing neuroscientific evidence, principles, and conceptual frameworks. Accordingly, it does not present new empirical experiments or participant-derived data.

Although the study principally adopts a neuroscientific perspective, it is informed by and remains in dialogue with relevant philosophical and psychological discussions. It is not intended, however, as a comprehensive philosophical or psychological analysis of mental causation. Since it does not systematically analyze every aspect of this matter, it does not aim to provide a final solution to the problem. Rather, its principal objective is to investigate the issue in detail from a neuroscientific vantage point, seeking to provide a new neuroscientific account and, possibly, a new neuroscientific explanation for it.

## 2 Study population and definitions

The present analysis is based on neuroscientific evidence and concepts derived primarily from studies of healthy, communicative, and cooperative adults—the population most capable of providing reliable reports and exhibiting observable effects of qualia and consciousness. Therefore, the working definitions, analyses, and conclusions presented here apply most directly to this group. These definitions largely follow those employed in a recent article on consciousness (Ukachoke, 2026) and are as follows:

### 2.1 Qualia (singular: quale)

In this study, the term “*qualia*” refers to *all and only those phenomena that appear phenomenally—manifesting what they are like*—*in the mind*. Accordingly, we can normally be aware of and experience them in a way that enables us to report both their occurrence and what they are like (based on Block, 1995; Koch, 2004; Kanai and Tsuchiya, 2012). For example, mental images, sounds, smells, emotions, and thoughts qualify as qualia by this definition because they appear phenomenally—manifesting what the images, sounds, smells, and so on are like—in our minds, enabling us to be aware of and experience them in the way that we can, under normal conditions, report their occurrence and what they are like.

According to current neuroscientific principles, because awareness and experiences of what qualia are like require functions of some neural circuit systems (Searle, 2000; Edelman, 2003; De Sousa, 2013; Purves and Platt, 2018; Kandel and Shadlen, 2021), qualia must appear phenomenally to those neural processing systems as well. Otherwise, such systems would lack the necessary substrate to generate awareness and experiences of that phenomenal appearance. Thus, neurologically, qualia (all and only those phenomena that appear phenomenally in the mind) are all and only those phenomena that appear phenomenally to some neural processing systems, especially those that generate awareness and experiences. Notably, this definition is grounded in neuroscientific principles and does not presuppose any metaphysical properties of qualia in advance.

### 2.2 Consciousness (adjective: conscious)

The term “*consciousness*” here refers to *awareness of what something is like* (based on Block, 1995; Chalmers, 1996, pp. 3–31; Koch, 2004). For instance, to be conscious of an image, sound, smell, emotion, or thought is to be aware of what that image, sound, smell, emotion, or thought is like, respectively. Such awareness is typically accompanied by an experience (Chalmers, 1995). Thus, in total, consciousness is both awareness and an experience of what something is like. Since “something” in this case manifests what it is like, it is a quale. Accordingly, *consciousness is awareness and an experience of what a quale is like*. This statement is supported by the observation that we cannot directly be aware of or experience what physical things or activities—such as an object, air vibration, molecules contacting our tongue or nasal mucosa—themselves are like. Instead, we can only be aware of and experience what their representations (in the forms of qualia, such as image, sound, taste, or smell qualia) are like. Moreover, generally, we are aware of and experience several qualia at the same time. For example, right now, the reader is aware of and experiences the image, sound, and smell qualia of the surroundings, as well as the thought quale in the reader’s mind. Therefore, in this study, *consciousness is awareness and experiences of what qualia are like*.

In this study, related terms—*conscious awareness* and *conscious experiences*—are also used with the following meanings: *conscious awareness* and *conscious experiences of some qualia* (such as image, sound, and taste) refer to *awareness* and *experiences, respectively, of what those qualia are like*. These two terms, when used together, will be shortened to *conscious awareness and experiences*.

Notably, consciousness by this definition is not, and does not consist of, other cognitive functions such as learning, problem-solving, decision-making, communication, or volitional motor control, although it normally accompanies these functions. That is, we normally have conscious awareness of and experience these cognitive activities, even though consciousness itself does not perform them—it merely functions to be aware of and experience them. This distinction is significant because consciousness and these other cognitive functions may have different causal efficacy.

Finally, because consciousness of something, such as awareness and experiences of what an image quale of a flower is like, manifests phenomenally in the mind, enabling us to know and experience what such consciousness is like and to tell that it has occurred, *consciousness is—based on the above definition of qualia—a type of quale*. Accordingly, this study will address the problem of whether and how consciousness and qualia in general, not just consciousness, exert physical effects. However, at times, for conciseness, only one will be discussed as a prototype.

### 2.3 Information

The term “*information*,” as used in this study, refers to *a non-material entity*^1^ *that consists of physically transmittable and causally effective content* (based on Bateson, 1972; Landauer, 1999; Floridi, 2009; Deutsch and Marletto, 2015; Dubrovsky, 2019; Krzanowski, 2020). For example, information on this page is non-material and consists of content (about term definitions) that is transmittable from the author to the page and then to the reader and is causally effective, causing specific patterns of dots and pixels on the page and certain cognitive activities (such as interpreting, analyzing, and remembering the definitions) in the reader. Similarly, information transmitted between neural circuits is non-material. It consists of content (something the sending neural circuit communicates to the receiving neural circuits), which is transmittable between neural circuits and causally effective, inducing the receiving neural circuits to execute certain activities.

## 3 Physical events occurring around the occurrence of a quale and consciousness

**Figure 1:**
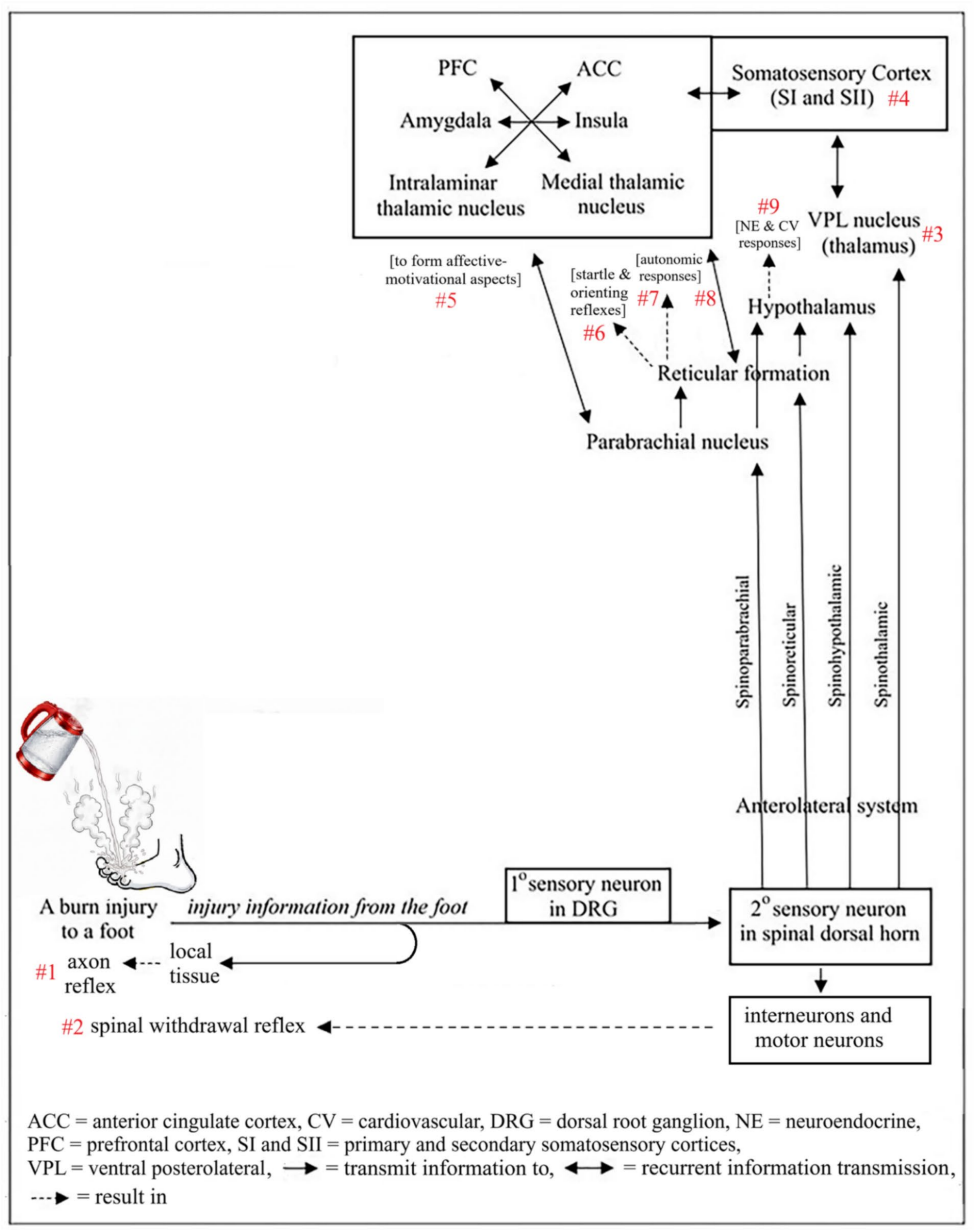
A schematic depiction of neural and behavioral events occurring before the emergence of a pain quale.

To assess whether qualia and consciousness exert physical effects, this study examines neural and behavioral events surrounding the emergence of a pain quale and its conscious awareness and experience in a typical scenario. A common example used to argue against the causal efficacy of qualia and consciousness is a case of a hand touching a hot stove, followed by withdrawal, vocalization, orienting behaviors, and other events. These responses are often attributed entirely to neural activity, without contribution from qualia or consciousness. To evaluate this notion, consider a similar case: A jug of hot water spills onto a person’s foot (Figure 1).

### 3.1 Peripheral nerves and primary sensory neurons

First, the burning stimulus is transduced into electrical signals by the foot skin’s nociceptors. Then, the signals are transmitted, in the form of action potentials, along the peripheral axons of primary sensory neurons toward their cell bodies in the dorsal root ganglion. After passing through the ganglion, the signals continue along the central axons of these neurons to synapse onto secondary sensory neurons in the dorsal horn of the spinal cord (Fitzpatrick and Mooney, 2018; Basbaum, 2021; Ambron, 2022).

Meanwhile, some impulses in the peripheral axons enter collateral branches of those axons and propagate back toward the injured site. These signals trigger the release of neurotransmitters, leading to vasodilation and other local inflammatory responses. The foot becomes red and swollen. This process is known as *the axon reflex* (Holzer, 1992; Yaprak, 2008).

### 3.2 Secondary sensory neurons and the local spinal cord

Most signals reach the secondary neurons and undergo initial processing there. Then, some are relayed to interneurons and subsequently to spinal motor neurons, which send signals to leg muscles, causing an abrupt withdrawal of the foot. This reflex is termed the spinal withdrawal reflex or nociceptive flexion reflex (Sherrington, 1910; Rhudy and France, 2007; Todd, 2010).

Other signals travel along the axons of the secondary neurons to higher brain centers. These axons first cross the spinal cord’s midline and then ascend in its anterolateral quadrant, forming the anterolateral system, which includes several ascending pathways (Willis, 2007; Todd, 2010; Fitzpatrick and Mooney, 2018; Amaral, 2021).

### 3.3 Ascending anterolateral pathways

Ascending signals are transmitted by several major tracts that subserve complementary functions:

*Sensory-discriminative processing*: Via the spinothalamic tract, signals reach the ventral posterolateral (VPL) thalamus and are relayed to primary and secondary somatosensory cortices. These cortical regions successively process the signals and generate the sensory-discriminative components of pain—its quality (e.g., burning, aching, stinging, stabbing, and lancinating), intensity, and spatial location. These pain components serve as substrates that will later be integrated with affective and motivational elements, provided by other pain pathways, into a unified, conscious perception of the pain (Fitzpatrick and Mooney, 2018; Amaral, 2021; Basbaum, 2021; Ambron, 2022, pp. 19–121).

*Affection-salience processing*: Via the spinoparabrachial and spinoreticulothalamic pathways, signals project to the parabrachial nucleus, hypothalamus, amygdala, insula, anterior cingulate cortex, and related limbic structures, generating emotional and motivational responses, such as astonishment, irritation, and aversion (Fitzpatrick and Mooney, 2018; Chiang et al., 2019; Amaral, 2021; Basbaum, 2021).

*Reflexive responses*: Via the spinoreticular pathways, some signals contribute to *the startle reflex*, which includes blinking, grimacing, vocalization, hunching, and arm flexion (Naysmith et al., 2021; Zhang et al., 2022), and to *the orienting reflex*, which involves a sudden cessation of ongoing activity, a shift of attention, head and eye movements toward the injured site, and transient cardiovascular changes (Kimmel, 1979; Pribram, 1979; Ranganath and Rainer, 2003).

*Autonomic and neuroendocrine responses*: Via the spinoreticular and spinohypothalamic pathways, signals are conveyed to the brainstem reticular formation and hypothalamus, eliciting autonomic changes (e.g., increased heart rate, blood pressure, vasoconstriction) and neuroendocrine responses (Willis and Westlund, 1997; Fitzpatrick and Mooney, 2018; Amaral, 2021; Basbaum, 2021).

Together, these pathways distribute nociceptive information across widespread neural systems, eliciting both neural and behavioral responses.

### 3.4 Pain matrix

Finally, when the pain signals from related cerebral processing areas—principally *the primary and secondary somatosensory cortices, insula, anterior cingulate cortex, and medial thalamus*, which are collectively known as the pain matrix—become functionally integrated through specialized, coordinated neural processing, the unified percept of pain arises (Bastuji et al., 2016; Fitzpatrick and Mooney, 2018; Garcia-Larrea and Bastuji, 2018; Ambron, 2022, pp. 188–235). At this point, the pain quale, which appears phenomenally—manifesting what the pain is like—to the processing systems of some neural circuits, occurs. Subsequently, consciousness, or conscious awareness and experience, of the pain quale is created by a certain group of those neural circuits.

The precise system of neural circuits and mechanisms by which processed pain signals become the phenomenal pain quale and by which consciousness of the pain quale is generated remains not definitively determined. Current proposed mechanisms include global workspace processing and broadcast (Baars et al., 2021), recurrent processing (Lamme and Roelfsema, 2000), predictive processing (Hohwy and Seth, 2020), information-integration-based processing (e.g., Orpwood, 2013; Balduzzi and Tononi, 2009; Tononi, 2012; Oizumi et al., 2014; Ukachoke, 2026), and recurrent representational processing involving “semblions” (neural representations of perception) in the Motivated Emotional Mind (MEM) model (Galus and Starzyk, 2020; Galus, 2022, 2025). The neural processes involved are likely complex, distributed, and functionally integrated across multiple brain regions. Moreover, these neural processes are typically dynamic, with continuously changing structures at the circuit, cellular, and subcellular levels for adaptation (Knudsen, 2013; Costandi, 2016, pp. 49–84, 125–156; Sanes, 2021; Banich and Compton, 2023, pp. 453–459). However, because these processes jointly contribute to the generation of a unified phenomenal experience of pain, the present study conceptualizes them collectively as “*the pain-quale-generating system*.” For a quale in general, this inclusive system is referred to as *a quale-generating system*. Similarly, the neural circuit system generating consciousness (conscious awareness and experience) of a quale—which is probably even more complex, more distributed, and more sophisticated—is collectively referred to as *the consciousness-generating system*.^2^

Crucially, although we presently still do not know the exact physical nature of the pain quale, except that it is non-material and generated by the pain-quale-generating system when this system has received the pain signals, we can analyze whether and how it has causal efficacy based on what happens in the neural circuit system after it occurs.

### 3.5 Downstream neural circuits

**Figure 2:**
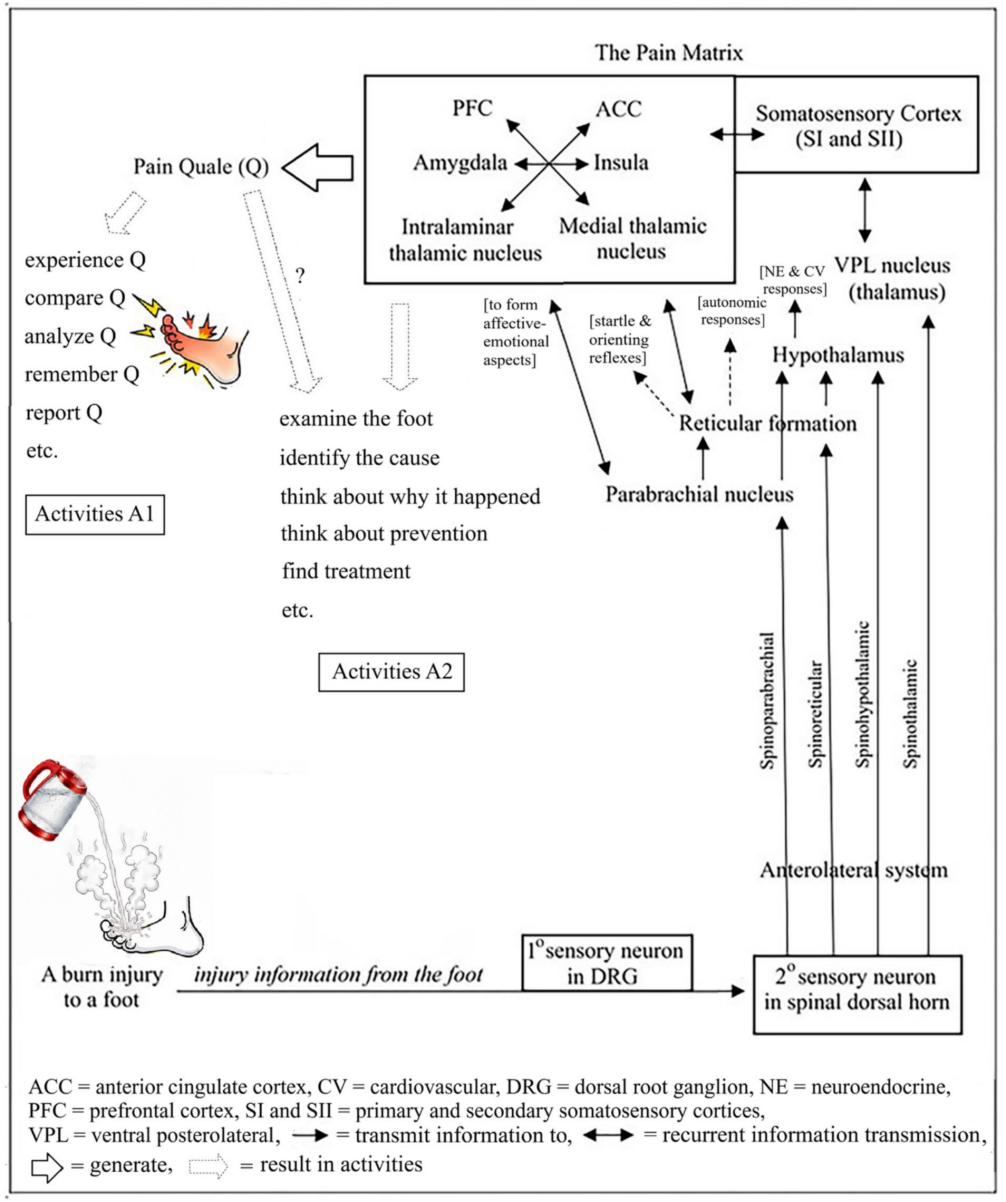
An extended schematic depiction demonstrating the generation of a pain quale by the pain-quale-generating system and subsequent neural and behavioral events in downstream neural circuits.

Following the pain quale’s occurrence, a variety of neural and behavioral activities related to it take place in the downstream neural circuits beyond the pain matrix, such as experiencing, comparing, analyzing, remembering, and reporting the pain quale (Figure 2). Also, higher-order, more complex activities involving multiple cognitive systems typically follow. These include examining the extent of the injury, identifying the causative object, reasoning about the event’s cause, considering ways to prevent similar incidents, and finding treatment for the pain.

## 4 The causal efficacy of qualia and consciousness

The preceding analysis demonstrates that numerous neural, autonomic, hormonal, and behavioral events occur in temporal proximity^3^ to the emergence of a pain quale and its conscious awareness and experience. These events are analyzed according to their temporal relation to the occurrence of the pain quale.

### 4.1 Events occurring before or in parallel with the generation of the pain quale

By the principle of temporal precedence—according to which a cause must precede its effect (Wallace, 1974; Andreas and Guenther, 2021; Palmer, 2023)—events occurring before or concurrently with the generation of the pain quale or its consciousness cannot be caused by them. These events include early neural processing, signal transmission along peripheral and central pathways, reflexes (e.g., withdrawal, startle, and orienting), and autonomic and neuroendocrine responses (labeled #1–#9 in Figure 1).

However, because some processes unfold over time, later stages of certain activities may still be modulated by the pain quale or its consciousness after they arise. Whether, and to what extent, such modulation is influenced by the phenomenal pain or consciousness of the pain cannot be concluded from this analysis and requires further investigation.

### 4.2 Events (activities) occurring after the pain quale generation

Events following the occurrence of the pain quale may, in principle, be caused or influenced by it. To emphasize their dynamic nature, they are referred to hereafter as activities. They are divided into two categories: those that explicitly involve the phenomenal pain quale (Activities A1) and those that do not (Activities A2) (see Figure 2).

#### 4.2.1 Activities A1

Activities in this category—such as experiencing, comparing, analyzing, remembering, and reporting the pain quale (Q)—explicitly involve the phenomenal features of the pain. According to neuroscientific principles, for such activities to occur, Q must be present in the neural system; otherwise, there would be nothing about Q for these activities to incorporate.

However, once Q has occurred in the neural system, it cannot plausibly cause or influence neural processing in a mechanistic, particle–force sense because, being a quale, it contains no particles and has not been found to exert any physical forces. This apparent incapability of Q may lead to the conclusion that Q is causally inefficacious and that all subsequent neural processing and the resulting activities about it are produced solely by physical interactions among neural components.

Nevertheless, the above account addresses only a mechanistic process—how neural processing unfolds through the force-based interaction of its particles under physical laws. It does not address what initiates and constrains that processing and determines the processing’s outcomes. Accordingly, although it can conclude that Q does not exert causal effects in the first respect, it does not establish that Q does not have causal influence in the latter respect. This latter aspect will now be examined.

First, based on current neuroscientific principles, for activities about Q, like Activities A1, to occur, Q must be processable by downstream neural systems; otherwise, there would be no neural processing about Q in those systems, and they would hence not be able to produce output activities about Q (e.g., Activities A1). Second, since neural processing normally involves information processing (Spruston et al., 2013; Abbott et al., 2021; Amaral, 2021; Shadlen and Kandel, 2021; Banich and Compton, 2023, pp. 14–17), Q must also comprise information about itself, such as information about what its phenomenal character is (i.e., burning, stabbing, throbbing, or another phenomenal character). Third, Q must be able to supply this information to the downstream neural circuits for processing. If not, those circuits would not have information about Q to process, and outputs about it would not be produced.

**Figure 3:**
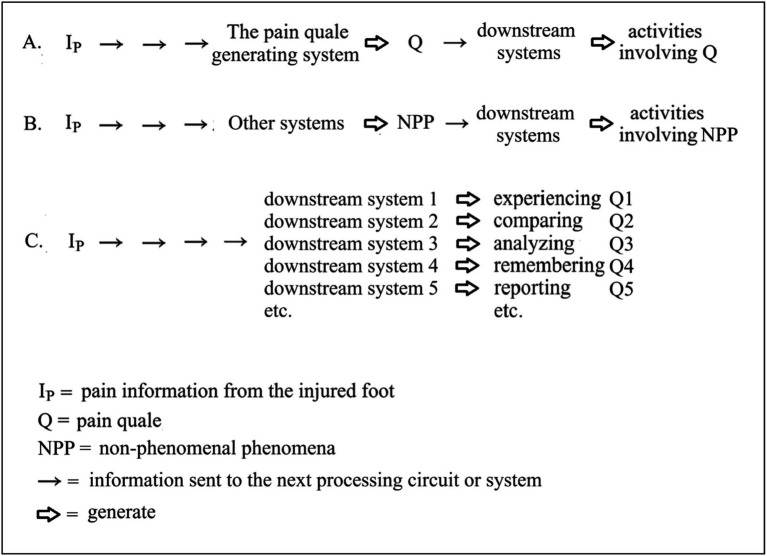
The pain information from the injured foot (I_P_) can be processed in three possible ways.

Because downstream neural processing about Q does occur, yielding various activities about Q, such as Activities 1, it can be logically inferred that Q has all three properties described above. Accordingly, when downstream neural systems receive Q’s information, the processing about Q initiates and proceeds in accordance with the received information. After processing this information, these systems generate activities involving Q—Activities A1—such as experiencing, comparing, and analyzing it (Process A in Figure 3). When the pain is burning, the resulting experiences, memories, reports, and other activities are correspondingly about burning; when it is stabbing, throbbing, or another phenomenal character, the outputs reflect that quality.

Crucially, if Q does not occur, there will be no information about it in the system. Without this information, neural processing about Q cannot be initiated, and Activities A1 cannot occur. Although non-phenomenal pain phenomena (NPP) may still be produced by some neural circuits and result in non-phenomenal processing and activities about them, such as reflexive, autonomic, or other responses, activities incorporating phenomenal content cannot be generated (Process B, Figure 3).

Because this case is representative, the same reasoning applies to qualia in general and to consciousness, which is a type of quale. If a quale or consciousness does not occur, there will be no information about the quale or consciousness in the neural system, and neural processing about it, as well as Activities A1, does not occur.

Thus, under normal conditions, a quale or consciousness is necessary for the initiation and structuring of neural processing about it; it also determines the content of the processing’s outputs. Importantly, the purely material systems consisting only of the quale- or consciousness-generating neural system and downstream systems, without generating the quale or consciousness first, cannot initiate these types of processing or produce these types of outputs, which are about the quale or consciousness, because they lack necessary information. Therefore, although qualia and consciousness themselves do not produce any outputs over and above what the neural system does, their presence or absence affects and makes a difference to that neural system. The neural system without them would not be as robust as the one with them—the part concerning processing and outputs about qualia or consciousness would be missing from the former system. On the other hand, importantly, a quale or consciousness by itself, lacking material constituents and physical forces, is unable to generate such processing and outputs, either. Rather, both parts must function together: The neural circuits implement the physical processing, while the quale or consciousness serves as the source of the indispensable phenomenal information for that processing.

Notes:

- One might argue that, even if Q does not occur, its information may still be generated in the neural system by some processes, thereby enabling awareness and experience of Q, as well as other activities about Q, as if Q had occurred. However, according to current neuroscientific understanding, except for pathological conditions—such as hallucinations, migraine auras, or epileptic auras—the nervous system would not normally generate information about something that does not occur within the system in a way that would make the brain process, think about, or experience that thing as occurring. Therefore, under normal conditions, which are the conditions studied in the present analysis, the above hypothetical scenario would not occur, and the Q’s occurrence is necessary for its information to be generated.
- Theoretically, the pain information from the injured foot (I_P_) may be transmitted directly to downstream systems via Route C (see Figure 3), and those systems each independently generate their specific output activities about the phenomenal pain. This process bypasses a prior generation of Q by the pain-qual generating system. However, this would require a comparable, complex, specialized system in every downstream circuit to separately generate a pain quale—Q1, Q2, Q3, and so on—for each circuit’s function. This possibility would involve significant redundancy in neural structures and processing and, hence, unnecessary increases in resource depletion and energy expenditure. Moreover, this scenario requires an explanation for how such separate systems can create pain qualia Q1, Q2, Q3, and other Q’s that are identical so that their outputs, such as experiencing, comparing, and analyzing the pain, do not involve different pain qualia with differing characteristics (e.g., all burning, not some burning, some stabbing, and some throbbing). Importantly, this scenario is not supported by current neuroscientific evidence and understanding, which is that a quale is generated by a certain neural system (a quale-generating system), as discussed in Section 3.4, and subsequently transmitted through the global workspace to various cognitive modules for the production of activities about the quale (Dehaene et al., 2014; Mashour et al., 2020; Baars et al., 2021). This study, therefore, holds that this possibility is not true.

#### 4.2.2 Activities A2

Activities in this category do not overtly involve phenomenal content. These include examining the injury, identifying its cause, or planning avoidance. Such activities could, in principle, occur without Q, driven solely by non-phenomenal signals, in a manner analogous to reflexive or automatic responses described in Section 4.1. This possibility is further supported by the observation that activities similar to Activities A2 can be implemented in robots or automated systems designed to respond adaptively to damaging insults, even though there is no evidence that they possess phenomena appearing phenomenally or awareness and experiences of such phenomena—i.e., qualia or consciousness—in their systems.^4^

However, because of complex interactions between various cognitive modules—both phenomenal and non-phenomenal—information about Q may also be transmitted from phenomenal modules, such as those for experiences, emotions, or memories of Q, to affect Activities A2. The extent of this influence remains an empirical question.

### 4.3 Summary

From the analysis, this study finds that, in the complex neural processing of pain stimuli, many neural and behavioral events of various types occur (Figures 1, 2), but qualia and consciousness do not cause or influence a large majority of them, which may lead to the notion that they are causally inefficacious and epiphenomenal.

However, although they do not cause events occurring before or during their emergence, and their influence on such events is limited or absent, this analysis finds that they do influence certain activities occurring after their emergence—specifically those produced by downstream systems and involving their phenomenal content. They exert such influence by providing the downstream processing with their phenomenal information, which is essential in initiating and structuring the neural processing about them and determining the processing outputs. Thus, although they do not act as mechanistic, force-based causes and are not independently sufficient, they play an integral and content-determining role.

Therefore, in this sense, even though qualia and consciousness themselves do not contribute any additional products than what the overall neural machinery does, they are not inert. Their presence or absence affects the neural system and makes a difference to it. Thus, if the non-mechanistic process described here can be considered a causal process, then qualia and consciousness can be considered causally efficacious, but only in the restricted and qualified but substantive sense discussed above. This study proposes that this non-mechanistic, information-based process is the basis for their causal roles in neural processing.

## 5 Discussion

The question of whether consciousness is causally efficacious—and, if so, how—has long been central to neuroscience and philosophy of mind, yet remains unresolved. The present study contributes to clarifying this issue by offering a neuroscientifically grounded analysis of the causal role of qualia and consciousness on neural and behavioral events occurring before, during, and after the emergence of a pain quale. Based current neuroscientific principles—especially that the neural system principally operates through information processing and that information is fundamentally what neural circuits process (Spruston et al., 2013; Abbott et al., 2021; Amaral, 2021; Shadlen and Kandel, 2021; Banich and Compton, 2023, pp. 14–17)—this study argues that, although qualia and consciousness themselves do not exert mechanistic particle–force effects, they nevertheless influence certain neural activities occurring after their emergence, specifically those involving their phenomenal content. They do so by providing their phenomenal information, which is essential for initiating, structuring, and determining neural processing about them, to downstream neural systems, as discussed above. The finding that qualia and consciousness affect neural processing can help address the three challenges raised in the introduction: It can explain how we can have knowledge and experience of consciousness, why they have been selected and preserved in the evolutionary process, and why consciousness occurs in us (e.g., because their effects can be beneficial to us).

A crucial question, however, is whether this account ultimately collapses into epiphenomenalism and whether qualia and consciousness themselves are causally efficacious. This problem remains because, obviously, it is only the neural components that mechanistically carry out the whole processing and produce the outcomes for the qualia and consciousness, which do not perform any mechanistic activity at all. This study finds that the answer depends on what is meant by “epiphenomenalism” and “causally efficacious.

- If “epiphenomenalism” means that *(a) all the mechanistic work is carried out solely by neural components with no mechanistic participation from qualia and consciousness* or *(b) qualia and consciousness do not contribute any outputs over and above what the neural system yields*, then qualia and consciousness can indeed be considered epiphenomenal. However, if epiphenomenalism means that *qualia and consciousness do not affect or influence neural systems at all*, then this analysis finds that qualia and consciousness are not epiphenomenal because *(a) they affect and influence neural systems in a non-mechanistic way* and (b) *although they do not produce any outputs over and above what the neural system does, some of those outputs (specifically, outputs about them) would not be produced in their absence*, as discussed in Section 4.2.1.
- If “causally efficacious” means “*able to mechanistically affect the causal process*,” then qualia and consciousness undeniably are causally inefficacious. But if “causally efficacious” means “*able to affect the causal process in some way, whether mechanistically or non-mechanistically*,” then this study finds that qualia and consciousness are causally efficacious but only in the restricted, information-providing sense described above.

Related topics, including non-mechanistic causation, are discussed next.

### 5.1 Dual-aspect naturalism

An important class of approaches relevant to the present study is dual-aspect naturalism (e.g., Russell, 1992; Strawson, 2006, 2008; Atmanspacher, 2012), including information-based dual-aspect theories of consciousness (e.g., Chalmers, 1995, 1996, pp. 284–287, 1997; Velmans, 2002, 2009). This framework exists in several forms, but in many versions the causal efficacy of consciousness is explained by regarding consciousness and the physical process as two aspects of the same underlying informational reality and denying consciousness as a separate causal entity in addition to the physical process.

In such accounts, the physical aspect carries the ordinary mechanistic causal relations described by neuroscience and physics, whereas the phenomenal aspect is not conceived as an additional force superimposed upon physical processes. Conscious experience is therefore considered causally efficacious because it is identical with, inseparable from, or constitutively related to the physically causal informational process itself. Accordingly, when a conscious state influences behavior or cognition, the causal process is understood as one integrated informational process viewed externally as neural causation and internally as conscious experience. In this way, consciousness is not regarded as existing outside the causal chain.

However, a persistent challenge for such approaches is that, if the physical description is already causally complete, it remains unclear what additional explanatory role phenomenality itself plays. This concern is closely related to the causal exclusion problem discussed by Kim (2005, 2007). Dual-aspect theorists typically respond that the phenomenal and physical are not two competing causes but two descriptions or aspects of one causally efficacious process. Critics, however, sometimes argue that this may merely redescribe the problem rather than fully resolve it: If the physical aspect alone is already mechanistically sufficient, why is the phenomenal aspect needed at all?

In relation to this analysis, the present study does not assert or deny that a quale is physically the phenomenal aspect of its associated information. Rather, it concludes more modestly that, based on current neuroscientific understanding—whatever a quale is—the information associated with the quale is not generated unless the quale itself occurs, and that the characteristics of the information constitutively depend on the phenomenal character of the quale (Section 4.2.1). Consequently, while downstream neural systems and the information implement the mechanistic processing, the quale itself, through its constitutive influence on the information, remains an indispensable, constraining, and content-determining factor within that causal process. This constitutes an explanation that differs subtly but significantly from those of traditional dual-aspect accounts and provides a more robust answer to why the phenomenal aspect is required: The quale is necessary because, without it, that information would not exist (no matter whether the quale is the phenomenal aspect of the information, identical to the information, or something else associated with the information), and the informational state required in that causal process would not occur. In this respect, the present account does not rely on the identity or dual-aspect relation between the phenomenal and the physical for the quale to have a causal role, but instead on the neuroscientific basis that indispensable information about the quale is not generated in the absence of the quale itself.

### 5.2 Motivated emotional mind (MEM) model

This section discusses a conceptually related example of a contemporary approach in which qualia and consciousness are understood as arising from distributed, recurrent, and embodied neural processes rather than from a sharply localized phenomenal center or an independent nonphysical mechanism. This newly emerging framework is the Motivated Emotional Mind (MEM) model proposed by Galus and Starzyk (2020) and further developed by Galus (2022, 2025). In this framework, neural representations called “semblions” are proposed as an organization of biophysical processes of association and transmission of neural excitations in hierarchical memory structures. Semblions are the essential neural substrate for, and the basis of, qualia and consciousness, but they are not identical to phenomenal experience. Instead, qualia and consciousness arise from specific patterns of semblion activity. Qualia arise when the system associates semblions (neural representations of perception) with emotional states resulting from internal body states (pain, pleasure, interoception, proprioception). However, the organism or artificial system must have a body or housing and a multimodal set of sensors recording its own states; without such embodiment, there can be no qualia.

Semblions underlie two distinct aspects of consciousness through separate neural processes:

- *Executive Consciousness* via Fast Forward Sweep (FFS)—bottom-up neural processes. The winning semblion activates motor fields, causing bodily action. This is automatic, deterministic, and causative. It is unconscious (though traditionally called “consciousness”).
- *Reporting Consciousness* via Recurrent Process (RP)—top-down feedback processes. The winning semblion sends retrograde signals to lower sensory fields, recreating sensory images (visualizations, memories). It is phenomenally conscious (phenomenal awareness, stream of consciousness, inner movie, etc.). It is epiphenomenal locally but modifies future states; thus, it is not completely epiphenomenal.

Notably, although information is fundamental in this framework, qualia and phenomenal consciousness are ontologically constituted not simply by information in the abstract, but by specific embodied and recurrently organized neural representations carrying sensory and emotional significance. Qualia are constituted by the integration or coupling of sensory representations with emotional/interoceptive representations into unified functional structures (“semblions”). Phenomenal reporting consciousness is constituted by top-down feedback processes that reactivate lower sensory layers, generating internally reconstructed perceptual sequences or an “inner movie.” This specific recurrent and embodied representational organization constitutes the conscious experience itself. Information alone (abstract, computational, or purely bottom-up) does not constitute consciousness. The neural architecture, direction of processing flow (especially top-down reactivation), and embodied coupling with emotional and bodily states are ontologically necessary components of phenomenal consciousness within this framework.

Thus, in this model, phenomenal experience does not arise from a distinct centralized quale-generating center or from an independent nonphysical source, but from recurrent reactivation and visualization of sensory representations embedded within distributed semblion structures involving multiple interacting neural layers and circuits. In this respect, the model shares important similarities with the present framework, as both accounts treat qualia and consciousness as arising from complex, distributed informational neural processes rather than from a single localized neural center or a separate nonphysical mechanism.

Both approaches also reject the notion that consciousness exerts additional force-like causal influence outside ordinary neural processing, and both treat consciousness as closely related to informational organization within neural systems rather than as a separate mechanistic entity. In both accounts, conscious experience influences future neural activity not through violation of physical causal closure, but through modification, restructuring, or constraining of subsequent neural states.

Thus, although the MEM model differs from the present account concerning the precise neural basis of phenomenality, both approaches reject strong epiphenomenalism and allow consciousness to exert a restricted, non-mechanistic influence on future neural processing.

### 5.3 Event- and property-epiphenomenalism

Epiphenomenalism exists in several forms. Not all of them deny the causal efficacy of mental states, events, or properties entirely; only specific strong forms do (Baysan, 2021). Primary examples of these strong forms include event (or token) epiphenomenalism and property (or type) epiphenomenalism (McLaughlin, 1989; Rostek, 2023; Walter, n.d.). Although their formulations differ, both maintain that consciousness and qualia play no causal role in producing physical effects. This study examines how these frameworks arrive at that position.

In the present case, as analyzed in Sections 3 and 4, numerous neural and behavioral activities occur in temporal proximity to the emergence of the pain quale. The two strong forms of epiphenomenalism hold that all such activities—whether preceding, concurrent with, or following the quale—are produced solely by neural processes. As discussed in Section 4.1, the present analysis concurs that events occurring before or concomitantly with the quale are caused by neural components alone. However, its conclusions about certain events occurring after the pain quale has been generated differ. This difference is now examined.

According to event epiphenomenalism, the pain-quale-generating system produces two outputs: a neural product and a pain quale. The latter is a distinct, non-material entity that accompanies the former but, unlike the former, does not influence neural processing. Under property epiphenomenalism, the pain quale is a property of the neural product yet remains causally inert. In both cases, the neural product enters the causal chain of neural processing and results in various activities—including the generation of experiences of the pain—while the pain quale itself contributes nothing. Hence, it is causally inefficacious.

However, at this point, it remains unclear whether this causally inert entity or property corresponds to the pain quale that appears phenomenally in our experiences. To avoid confusion, it will be referred to here as *the hypothetical pain quale* unless it can be shown to be identical to the experienced pain quale.

**Figure 4:**
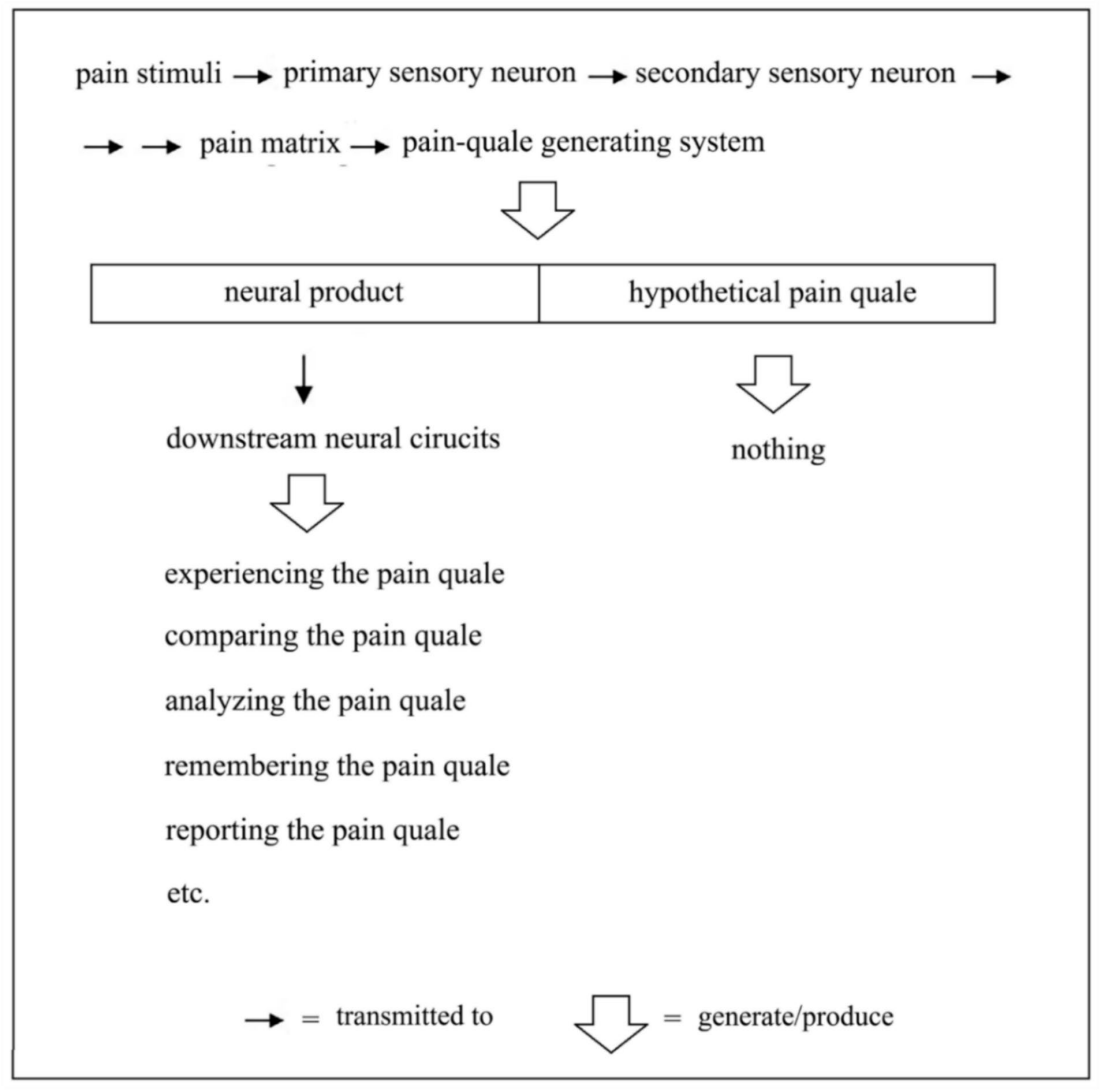
The pain-quale-generating system generates a neural product and a “hypothetical pain quale,” with subsequent production of several outputs about the pain quale from the neural product.

To determine whether the hypothetical pain quale appears phenomenally in the mind and is therefore the quale that we are aware of and experience, consider what occurs after it is generated (Figure 4).

After the neural product and the hypothetical pain quale are generated by the pain-quale-generating system, the neural product is transmitted to downstream neural circuits and processed there. Subsequently, those circuits generate activities such as experiencing, comparing, analyzing, remembering, and reporting the pain. Because these activities explicitly concern what the pain feels like, the neural product must carry information specifying the phenomenal character of the pain to those circuits.

By contrast, according to the two strong forms of epiphenomenalism, the hypothetical pain quale—whether conceived as a separate entity or as a property—plays no role in this process. It just accompanies the neural product but neither initiates nor participates in neural processing and contributes nothing to the resulting activities, including awareness or experience. Consequently, it does not give rise to awareness or experience of itself.

Because it is not experienced, the hypothetical pain quale cannot be the pain quale that appears in our minds and motivates the present inquiry. Thus, its causal inefficacy does not entail that the experienced pain quale is also causally inefficacious.

In relation to the present framework, this study does not posit that the pain-quale-generating system produces two outputs, as both strong forms of epiphenomenalism do. Instead, it proposes that the generating system yields one unified product: the pain quale. Whatever it’s ultimate physical nature may be (discussed in the next section), it is not causally inert. It is the source of the essential phenomenal information that the neural product carries to downstream systems for processing, which subsequently generate experiences about it. It is this experienced quale—not the hypothetical one—that constitutes the explanandum of mental causation.

In summary, strong epiphenomenalism identifies the experienced pain quale with a causally inert entity—the hypothetical pain quale. However, the present analysis finds that this identification is not supported. The experienced pain quale gives rise to awareness and participates in the generation of activities about itself, whereas the hypothetical pain quale does not. They are therefore not identical. While the hypothetical pain quale is causally inefficacious, the experienced pain quale is causally efficacious in the qualified sense developed here.

### 5.4 The physical nature of qualia and consciousness

A natural question arising from the preceding analysis is: What is the physical nature of causally efficacious qualia and consciousness, and how do they transfer their information to downstream neural circuits? From earlier discussions, they can be characterized as non-material entities generated by specific neural systems and capable of influencing neural processing without themselves exerting physical forces. However, their precise physical nature remains open: Are they novel entities outside the current physical framework, or are they known physical entities, yet unrecognized as qualia or consciousness?

Although the present study does not aim to provide a complete answer, some constraints can be identified. As discussed in Section 4.2.1, after information about a pain quale is generated by the pain-quale-generating system and subsequently processed by downstream neural circuits, activities such as experiencing that pain occur. For such experience to arise, this information must appear phenomenally to the processing system of the experience-generating system; otherwise, that system would lack a phenomenal basis for generating the experience of the phenomenal pain.

This observation suggests that the pain quale is closely related to its information, insofar as the circuit treats the information as constituting the phenomenal content of the experience. A parsimonious possibility, therefore, is that the pain quale is identical its phenomenal information, which is the phenomenal aspect or counterpart of its physical information, as discussed in Section 5.1. Since information is a physical entity,^5^ this possibility is within the boundaries of the current scientific framework. This possibility can also parsimoniously explain how the pain quale can transfer its information to relevant neural circuits: Information is precisely what neural circuits can naturally receive, as well as compute and propagate.

Another possibility is that the quale consists of this information together with an additional, as yet unspecified component that enables or enhances this information’s transmission and phenomenal manifestation. A further possibility is that the quale is an entirely distinct entity unrelated to this information but capable of augmenting the involved circuits in receiving the information and forming experiences and other activities concerning the information by some unknown mechanisms. However, these two alternatives introduce novel elements and thus are less parsimonious.

Notably, the concept that the physical nature of qualia and consciousness is intrinsically tied to information is broadly compatible with several existing frameworks, including the Dual-Aspect Theory of Information (Chalmers, 1995), Integrated Information Theory (Tononi, 2012), the proposal in “Information and the Origin of Qualia” (Orpwood, 2017), the Information Closure Theory of Consciousness (Chang et al., 2020), the Motivated Emotional Mind (MEM) (Galus and Starzyk, 2020; Galus, 2022, 2025), and The Basic Theory of the Mind (Ukachoke, 2024, pp. 87–105). A full evaluation of these frameworks, however, requires extensive treatment beyond the scope and spatial constraint of this paper. Thus, the reader is referred to the cited literature.

Importantly, the conclusions of the present analysis do not depend on resolving this issue. Whether qualia and consciousness are identical to specific forms of information, involve additional components, or possess a different constitution altogether, and whatever the exact mechanism of information transfer may be, the conclusions of Section 4.2.1 remain unchanged. These conclusions rest on current neuroscientific principles and empirical observations about their role in neural processing: (1) activities involving a quale or its consciousness occur only after it arises and do not occur in its absence, and (2) the content of these activities varies systematically with its phenomenal character. These features indicate that qualia and consciousness exert causal influence on such activities regardless of their precise physical nature and mechanism of information transfer.

### 5.5 Non-mechanistic causation

The non-mechanistic causal influence attributed here to qualia and consciousness resembles the role played by information in many causal processes. Information is non-material and does not exert any physical force, yet it is indispensable in initiating and structuring causal chains about it. For example, the information conveyed by a written text or spoken instruction can initiate complex causal processes in a reader or listener, shaping thoughts and actions, even though it consists of no particles and exerts no force. This form of influence has been described as information causation (Dubrovsky, 2019). In such cases, physical systems perform all mechanistic work, but only in the presence of relevant information. Thus, information and the physical systems that process it must function together for causal processes to be initiated and to produce outcomes specific to that information. This joint functioning parallels the role of qualia and consciousness operating together with neural circuits, as described above.

The idea that causation need not be exclusively mechanistic is not new. The assumption that all causation—particularly causal interaction involving the mind—must be explicable in terms of direct push-and-pull mechanisms has long been challenged in the philosophical literature. Richardson (1982), for example, argues that the traditional “scandal” of Cartesian interactionism rests largely on the assumption that all causation must conform to classical push-and-pull models. Rejecting this assumption weakens standard objections to non-mechanistic accounts of mental causation without committing one to substance dualism or violations of physical law.

Related distinctions have been developed in contemporary philosophy of science. Jackson and Pettit (1990), for instance, distinguish causal efficacy from causal relevance, arguing that higher-level properties can structure or program lower-level processes without exerting physical force. Properties such as fragility or knowledge do not themselves perform physical work; rather, they constrain how physical processes unfold—for example, how glass responds to mechanical stress or how a person behaves under particular circumstances—and thereby help explain why certain outcomes occur instead of others.

Similarly, Woodward’s (2003) interventionist account characterizes causation in terms of stable counterfactual dependence under intervention, rather than in terms of underlying mechanisms. Menzies and List (2010) likewise describe causation in terms of difference-making relations: Higher-level properties qualify as causes when variations in them systematically produce variations in outcomes, even though lower-level processes implement the causal chain.

From this perspective, qualia and consciousness can be understood as higher-level, non-mechanistic factors that make a difference to which neural processes are initiated and to what outputs are produced. In Jackson and Pettit’s terms, they are causally relevant without being mechanistically efficacious; in Woodward’s and Menzies and List’s terms, they stand in stable difference-making relations to their downstream neural and behavioral effects.

Crucially, in the present analysis, this non-mechanistic causal efficacy is mediated by phenomenal information. Qualia and consciousness are necessary for the generation of their information, which is required for initiating, structuring, and determining neural processing about them. It is plausible that the causal relevance of higher-level factors in general may likewise derive from the information they contribute to lower-level physical systems. However, this generalization remains speculative and lies beyond the scope of the present study.

The present account also relates to the causal exclusion problem in philosophy of mind, particularly as formulated by Kim (2005, 2007). If physical neural processes are causally sufficient for behavior and cognition, it may appear that there is no remaining causal work for consciousness or qualia to perform. The present framework attempts to address this problem not by proposing that qualia exert additional force-based causal influence alongside neural processes, but by treating them as higher-level informational and constraining factors within neural causal organization itself. In this view, qualia and consciousness do not compete with physical neural causes; rather, they function together with the physical neural processes by providing essential phenomenal information required for the processing.

Interestingly, one parsimonious theoretical possibility, discussed in the previous section, is that Q may be identical to its phenomenal information, physically instantiated within neural systems. Since information, in this framework, is regarded as a physical entity (see Footnote 5), such a view may partially address Kim’s concern that mental causation requires some form of physical realization or reduction in order to avoid epiphenomenalism. This is an interesting theoretical possibility that this study observes and raises for consideration because it may yield some clues to address the present problem. However, the study itself does not directly analyze this matter or assert that phenomenal facts (like qualia) have been conclusively reduced to non-phenomenal physical facts (like informational structure that is physically realized). A full resolution of this metaphysical issue lies beyond the scope of the present study. Rather, the present analysis aims primarily to clarify how qualia and consciousness may participate in neural causal processes while remaining consistent with physical causal closure.

Regarding related ideas proposed by Rosenberg that phenomenal properties provide “causal significance” by constraining or determining the resolution of indeterminate possibilities (Rosenberg, 2004), the present framework proposes similar concepts that qualia and consciousness function as ineliminable determinants within the causal chains concerning them. Although they are not conceived as separate mechanistic forces acting in violation of physical principles, neural processing outputs concerning a conscious experience depend on both the occurrence and phenomenal content of that experience. In this sense, conscious experience is not epiphenomenally redundant but constitutes an indispensable factor in the organization and progression of the relevant neural-information processes. The causal efficacy of consciousness in the present framework therefore does not arise from introducing additional force-like interactions into physics but from the fact that conscious experience itself constitutes the determining informational content that shapes subsequent neural processing and behavioral outputs related to that experience. Although Rosenberg’s metaphysical framework differs substantially from the present neuroscientific account, his notion that phenomenal properties possess “causal significance” through their non-redundant determining role bears conceptual similarity to the present proposal.

In conclusion, expanding the explanatory framework to include non-mechanistic, informational causation provides a more holistic and accurate account of mental causation. Non-mechanistic causation does not replace mechanistic causation; both complement each other. Mechanistic causation explains how physical processes unfold through force-based interactions; non-mechanistic causation explains what is needed for those processes to initiate and why they take the specific, content-dependent form and produce certain content-bearing outputs they do. Together, both types of causation offer a more complete and scientifically cohesive picture of how subjective experience seamlessly participates in the causal architecture of the physical brain without violating the causal closure of the physical.

### 5.6 The ultimate cause

One may argue, however, that because the pain quale is generated by the pain-quale-generating system, it is actually this neural system—not the pain quale—that exerts causal efficacy on downstream processing, and it is the ultimate cause of downstream processing outputs.

Regarding the first point, the present analysis suggests that both exert causal efficacy. The pain-quale generating system alone cannot exert causal effects on downstream processing about the pain quale because the processing requires information about the pain quale itself. Therefore, the pain-quale generating system must generate the pain quale first—it cannot bypass the generation of this essential element. Accordingly, the quale-generating system exerts its causal efficacy in this process precisely by generating the pain quale, which then exerts influences on downstream processing by providing its information, required for downstream processing concerning it.

Regarding the ultimate cause, although it is true that the pain-quale generating system can be considered a higher-level cause than the pain quale, the question addressed in this study is not what serves as the ultimate origin of the causal chain and its outputs. Rather, the question is *whether qualia and consciousness are causally efficacious within the processes in which they occur*. In this respect, and in the qualified sense specified earlier, they do influence neural processing about them, irrespective of the fact that they are themselves generated by some neural systems.

In fact, appealing to ultimate origins does not resolve the present problem but merely displaces it. Any such appeal leads to an indefinite regress: Neural circuits and their states arise from prior physical structures and states, which in turn derive from earlier ones—extending through the individual’s development, human evolutionary history, the emergence of life, and ultimately the origin of the universe. Invoking ultimate causation, therefore, does not solve the problem of whether qualia and consciousness themselves are causally efficacious.

To recap, the primary question here is whether qualia and consciousness themselves make a difference within the causal processes in which they occur. The present analysis indicates that they do, whatever the ultimate cause of the outcomes is.

### 5.7 Generalizability

As noted in Section 2, because this study is based on neuroscientific evidence and concepts derived primarily from studies of healthy, communicative, and cooperative adults, the findings and conclusions in this analysis apply most directly to this group. Presently, however, there is no neuroscientific evidence that the causal mechanisms proposed in this study—namely, that qualia and consciousness enable the generation of phenomenal information required for initiating and structuring neural processing about them and for determining the content of its outputs—would fundamentally differ across age, neurological condition, or mental state. Accordingly, the findings as well as the conclusions are likely to apply more broadly, including to infants, non-communicative adults, and non-cooperative individuals. Nevertheless, such generalization remains provisional and can be confirmed more definitively only when objective methods for detecting qualia and consciousness—independent of report or cooperation—become available.

A further limitation concerns the scope of the analysis. This study focuses specifically on qualia and consciousness—that is, phenomenal mental events that manifest what they are like. It does not directly address other mental states, such as occurrent propositional attitudes (e.g., decisions, beliefs, and desires), insofar as these states may lack phenomenal character. If such states do not constitute qualia, their causal efficacy is not supported by the present analysis. If, however, they possess a phenomenal aspect, then they may be causally efficacious in the restricted sense described here.

Even in that case, the causal role identified pertains specifically to their phenomenal character. For example, a conscious decision to act may be causally efficacious in enabling activities that incorporate its phenomenal character—such as experiencing, remembering, or reporting *what consciously making the decision is like*—but this does not establish that a conscious decision directly causes the resulting motor action. Whether conscious decisions initiate voluntary movements or other actions is a distinct question, as explored in Libet-style paradigms (e.g., Libet et al., 1983; Wegner and Wheatley, 1999; Trevena and Miller, 2002; Matsuhashi and Hallett, 2008; Soon et al., 2008) and remains a topic of ongoing debate (see, for example, Bonicalzi and Haggard, 2019; Kozuch, 2020; Braun et al., 2021).

Accordingly, the conclusions of this study should not be extended uncritically to non-phenomenal mental states. Their causal roles require independent empirical and theoretical investigation.

## 6 Conclusion

This study presents a detailed theoretical neuroscientific analysis of the causal efficacy of consciousness and offers a neuroscientific explanation for it. The study analyzes neural and behavioral events occurring before, during, and after the emergence of a pain quale in a typical scenario. Based on current neuroscientific principles, the analysis concludes that many such events are not caused or influenced by qualia or consciousness, whereas others are initiated or influenced by them, at least in part. Therefore, it proposes the conclusion that qualia and consciousness are not causally inert.

The study finds that their influence does not operate through mechanistic particle–force interactions. Rather, they function as indispensable factors that initiate and structure the causal chain of neural processing about them and determine the content of its outputs. They perform this role by providing their essential phenomenal information to these processes and act as higher-level factors that stand in constitutive and stable counterfactual relations to their effects.

Nevertheless, they are not independently sufficient: They cannot exert influence in isolation but must operate in conjunction with neural circuits. Conversely, neural circuits alone, in the absence of the qualia or consciousness, are insufficient to produce neural processing about them, either. Thus, both components—the quale or consciousness and the associated neural circuitry—must be present and function together for the causal chain to be complete and capable of producing outputs concerning that qualia or consciousness.

Consequently, the question of whether consciousness is causally efficacious does not permit a simplistic, unqualified answer. Moreover, its causal status is fundamentally conditional, depending on how an individual framework defines the boundaries of causation itself. On the one hand, if causation is defined as the capacity to exert physical force or manipulate matter mechanistically, then qualia and consciousness must be classified as causally inefficacious and epiphenomenal. On the other hand, if causation encompasses the capacity to systematically constrain, program, and alter the outcomes of a physical system in whatever way, then qualia and consciousness are genuinely causally efficacious and not epiphenomenal.

While this study recognizes that the ultimate choice between these frameworks depends on one’s philosophical commitments, it firmly demonstrates that consciousness is not inert and not an empirically redundant byproduct. And, if viewed as causally efficacious, it operates in a qualified, non-mechanistic, and information-providing capacity. One virtue of this account is that it offers a parsimonious explanation for the causal role of qualia and consciousness: It explains how qualia and consciousness—despite lacking material mass, physical forces, or the capacity for mechanical interaction—can affect and shape the physical world without violating the causal closure of the physical universe. Together with existing theoretical frameworks, it may contribute to a more comprehensive understanding of this crucial matter.

Recognizing that qualia and consciousness can influence the physical world in this specified and limited, yet substantive, sense—and that they do so through their phenomenal information—may help clarify related questions, including their physical nature, the hard problem of why they occur, and their evolutionary role. Although these issues lie beyond the scope of the present study, the framework proposed here may assist in addressing these and other associated problems.

## Data Availability

The original contributions presented in the study are included in the article/supplementary material, further inquiries can be directed to the corresponding author.

## Footnotes

1 In this study, “*material*” means “*composed of elementary particles with mass* (e.g., electrons, muons, and neutrinos)” and “*non-material*” means “*not composed of elementary particles with mass*.” Crucially, “non-material” does not mean “non-physical.” For example, electromagnetic waves, electrical fields, and magnetic fields are non-material, but they are physical, governed by known physical laws.

2 The terms “the pain-quale-generating system,” “a quale-generating system,” and “the consciousness-generating system” here are intended only as functional and inclusive working terms encompassing the distributed neural systems proposed in existing models of phenomenal consciousness, such as those exemplified in this section. They are used as working terms in this study and not proposed as new standard terms in neuroscience or philosophy.

3 According to current neuroscientific evidence, conscious perceptual access to painful stimuli typically depends on late-stage, large-scale cortical processing occurring approximately 300–500 ms (milliseconds) after a stimulus. This conscious access is characterized by several neurophysiological events, including the P300 response and increased large-scale gamma-band oscillatory synchronization across distributed cortical regions (Melloni et al., 2007; Gaillard et al., 2009; Dehaene and Changeux, 2011; Dehaene, 2014). Moreover, behavioral reaction times to painful stimuli are commonly reported within a roughly similar range, approximately 350–450 ms (for example, see Arendt-Nielsen and Bjerring, 1988). Accordingly, the neural, autonomic, hormonal, and behavioral events discussed in the present analysis occur within this general time window. Their temporal relations to one another—and particularly to the emergence of the phenomenal pain experience—are therefore expected to lie within less than 500 ms.

4 This is because such phenomena and their related awareness and experiences, being new activities in those systems, would require additional specialized circuits and processes to systemically generate and manage them. But, presently, we do not know how to construct such circuits with such processes and have not included them in the processing systems of today’s robots and other automated machines (Dehaene et al., 2017; Ukachoke, 2024, pp. 166–170).

5 Information is physical in the sense that it is always tied to a physical object (Landauer, 1996, 1999; Floridi, 2005; Deutsch and Marletto, 2015; Dubrovsky, 2019; Krzanowski, 2020), which is under physical laws. Accordingly, the laws governing information, although not simple physical laws (Wang, 2022), are derivable from them (Deutsch and Marletto, 2015). Moreover, some aspects of it can be quantified mathematically in various ways (e.g., see Shannon, 1948; Landauer, 1961; Kolmogorov, 1968), very much like physical entities in general.
